# Visceral adipose tissue is associated with occult synchronous peritoneal metastasis in colorectal cancer

**DOI:** 10.1515/pp-2024-0030

**Published:** 2025-04-24

**Authors:** Bei-bei Zhang, Han-bing Xie, Ping-ping Liu, Le Liu, Xue-meng Li, Lin Zhao, Guang-yu Wang, Rui-tao Wang

**Affiliations:** Department of Endoscope, 91631 Harbin Medical University Cancer Hospital, Harbin Medical University, Harbin, Heilongjiang, China; Department of Internal Medicine, 91631 Harbin Medical University Cancer Hospital, Harbin Medical University, Harbin, Heilongjiang, China; Department of Gastrointestinal Medical Oncology, 91631 Harbin Medical University Cancer Hospital, Harbin Medical University, Harbin, Heilongjiang, China

**Keywords:** colorectal cancer, visceral adipose tissue, synchronous PM

## Abstract

**Objectives:**

Synchronous peritoneal metastasis (PM) represents an advanced stage of colorectal cancer (CRC), indicating extensive tumor spread. Visceral adipose tissue (VAT) has been linked to cancer development and progression. This study aims to explore the relationship between VAT and occult synchronous PM in CRC patients before surgery.

**Methods:**

We enrolled 681 CRC patients, with 419 in the primary cohort (356 non-metastatic and 63 with PM) and 262 in the validation cohort (239 non-metastatic and 23 with PM). Clinical characteristics and laboratory measurements were collected prior to surgery. Adipose and muscle tissues were delineated on preoperative contrast-enhanced computed tomography (CT) images. The association between visceral adipose tissue and synchronous PM was assessed using univariate and multivariate logistic regression analyses.

**Results:**

In the primary cohort, 419 patients were diagnosed with CRC, with 63 presenting with PM. Patients with PM had higher visceral adipose tissue index (VATI) levels compared to those without PM. Additionally, there was a trend towards increased PM incidence with elevated VATI. Multivariate logistic regression analysis confirmed that higher VATI was independently associated with PM. These findings were consistent in the validation cohort.

**Conclusions:**

VATI is an independent risk factor for occult synchronous PM in patients with CRC.

## Introduction

Colorectal cancer (CRC) is the third leading cause of cancer-related mortality globally, with synchronous peritoneal metastasis (PM) occurring in 1–13 % of patients at diagnosis and conferring a poor prognosis (median survival: 4–7 months) [1], [2], [3], [4], [5], [6]. Current detection methods, including CT imaging (11 % sensitivity for lesions <5 mm) and tumor biomarkers, lack reliability for early-stage PM, underscoring the urgent need for improved markers [7], 8].

Obesity-driven dysregulation of visceral adipose tissue (VAT) may play a pivotal role in metastatic progression. Excess VAT secretes proinflammatory adipokines that foster chronic inflammation and tumor proliferation [9]. Conversely, CRC cells reprogram adjacent adipocytes into a tumor-supportive phenotype, enhancing lipolysis and aerobic glycolysis to fuel metastatic spread [10], [11], [12]. While VAT is implicated in CRC’s tumor microenvironment, its specific association with synchronous PM remains unexplored.

This study investigates VAT as a potential biomarker for synchronous PM in CRC, aiming to inform early detection strategies and therapeutic targeting of adipose-driven metastasis.

## Materials and methods

### Patient characteristics

We conducted a retrospective study involving CRC patients who underwent contrast-enhanced CT and radical resection from January 2021 to December 2021. Patients were recruited from two tertiary hospitals: Harbin Medical University Cancer Hospital and the Second Affiliated Hospital of Harbin Medical University. Postoperative pathological reviews of surgically resected specimens by pathologists were used to diagnose PM.

The inclusion criteria for the study were: (1) all patients had a pathological diagnosis of CRC; and (2) all patients underwent radical resection of CRC. Exclusion criteria included: (1) the presence of multiple primary cancers; (2) receipt of preoperative neoadjuvant treatment; and (3) the unavailability of clinicopathologic data (Figure S1).

The collected clinicopathological parameters comprised age, gender, body mass index (BMI), tumor location, histological type, tumor stage (T stage), nodal stage (N stage), carcinoembryonic antigen (CEA), carbohydrate antigen 19‐9 (CA19-9), hypertension, diabetes, and albumin levels. TNM staging was updated according to the eighth edition of the American Joint Committee on Cancer (AJCC) Staging Manual.

The study received approval from the Ethics Committee of Harbin Medical University Cancer Hospital and the Second Affiliated Hospital of Harbin Medical University. The requirement for informed consent was waived due to the retrospective nature of the study.

### Adipose tissue imaging analysis

Adipose tissue was assessed using preoperative CT scans. The quantification of visceral and subcutaneous adipose tissues was performed at the L3 vertebral level employing Image J software version 1.53a (Wayne Rasband, National Institutes of Health, USA). For subcutaneous adipose tissue, Hounsfield Units (HU) thresholds ranged from −190 to −30. For visceral adipose tissue, the radiodensities were set between −150 and −50 HU [13], 14]. The visceral adipose tissue index (VATI) and subcutaneous adipose tissue index (SATI) were derived by normalizing the respective adipose tissue areas to the square of the patient’s height [12], 15]. The visceral adipose tissue index (VATI) and subcutaneous adipose tissue index (SATI) were derived by normalizing the respective adipose tissue areas to the square of the patient’s height.

### Statistical analysis

Statistical analyses were conducted using MedCalc version 15.0 and SPSS version 26.0 (SPSS Inc., Chicago, IL, USA). Continuous variables were expressed as means and standard deviations for normally distributed data or as medians and interquartile ranges for non-normally distributed data. Comparisons between groups were made using Student’s *t*-tests or Mann-Whitney U tests as appropriate. Categorical variables were analyzed using Chi-square tests. The optimal cut-off value for VATI in predicting PM was established using receiver operating characteristic (ROC) curve analysis. Risk factors for synchronous PM were initially identified through univariate analyses. Variables showing a significant association with synchronous PM (p<0.10) were further examined using a multivariate logistic regression model. A stepwise backward elimination method was applied, with variables required to have a p-value of less than 0.05 to enter the model and less than 0.10 to be removed. A p-value of less than 0.05 was considered statistically significant.

## Results

This study included 419 CRC patients (mean age: 57.0 ± 10.9 years), among whom 63 (15.0 %) were diagnosed with synchronous PM. Baseline clinicopathological analysis revealed significant differences between PM and non-PM groups in the primary cohort for age, albumin levels, histological type, T stage, N stage, CEA, CA19-9, and VATI. In the validation cohort, tumor location, histological type, CEA, CA19-9, albumin, and VATI also differed significantly between groups (Tables 1 and 3).

**Table 1:** The characteristics of CRC patients according to PM status in the primary cohort.

Variables	Total n=405	PM (+) n=49	PM (−) n=356	p-Value
Age, years	57.0 ± 10.8	60.9 ± 13.4	56.5 ± 10.3	0.032
Gender, %				
Male	234 (57.8)	31 (63.3)	203 (57.0)	0.407
Female	171 (42.2)	18 (36.7)	153 (43.0)	
Tumor location, %				
Right colon	93 (23.0)	14 (28.6)	79 (22.2)	0.319
Left colon + rectum	312 (77.0)	35 (71.4)	277 (77.8)	
Histological type, %				
Adenocarcinoma	336 (83.0)	30 (61.2)	306 (86.0)	<0.001
Mucinous/Signet ring cell	69 (17.0)	19 (38.8)	50 (14.0)	
T stage, %				
T1–T3	371 (91.6)	34 (1.6)	337 (94.7)	<0.001
T4	34 (8.4)	15 (98.4)	19 (5.3)	
N stage, %				
N0	245 (60.5)	11 (22.4)	234 (65.7)	<0.001
N1/N2	160 (39.5)	38 (77.6)	122 (34.3)	
CEA, ng/mL				
≤5	264 (65.2)	22 (44.9)	242 (68.0)	0.001
>5	141 (34.8)	27 (55.1)	114 (32.0)	
CA19-9, U/mL				
≤37	348 (85.9)	29 (40.8)	319 (89.6)	<0.001
>37	57 (14.1)	20 (59.2)	37 (10.4)	
Hypertension				
Yes	88 (21.7)	15 (30.6)	73 (20.5)	0.108
No	317 (78.3)	34 (69.4)	283 (79.5)	
Diabetes				
Yes	43 (10.6)	7 (14.3)	36 (10.1)	0.374
No	362 (89.4)	42 (85.7)	320 (89.9)	
BMI, kg/m^2^	24.0 ± 3.3	24.6 ± 3.1	23.9 ± 3.4	0.128
Albumin, g/L	41.5 ± 4.3	38.8 ± 4.8	41.9 ± 4.1	<0.001
Skeletal muscle area, cm^2^	128.9 ± 31.0	130.0 ± 35.5	128.8 ± 30.4	0.800
Skeletal muscle index, cm^2^/m^2^	46.3 ± 9.0	47.0 ± 9.6	46.1 ± 8.9	0.531
Visceral adipose tissue area, cm^2^	114.9 ± 67.0	157.3 ± 65.1	109.0 ± 65.2	<0.001
Visceral adipose tissue index, cm^2^/m^2^	41.4 ± 23.7	57.4 ± 23.2	39.3 ± 23.0	<0.001
Subcutaneous adipose tissue area, cm^2^	134.9 ± 61.8	136.9 ± 59.9	134.6 ± 62.1	0.812
Subcutaneous adipose tissue index, cm^2^/m^2^	49.4 ± 24.2	50.4 ± 23.6	49.3 ± 24.3	0.762

CRC, colorectal cancer; PM, peritoneal metastasis; CEA, carcinoembryonic antigen; carbohydrate antigen 19-9; BMI, body mass index; T stage, tumor stage; N stage, nodal stage; CA19-9, carbohydrate antigen 19‐9.

### Association between VATI and PM

**Figure 1:**
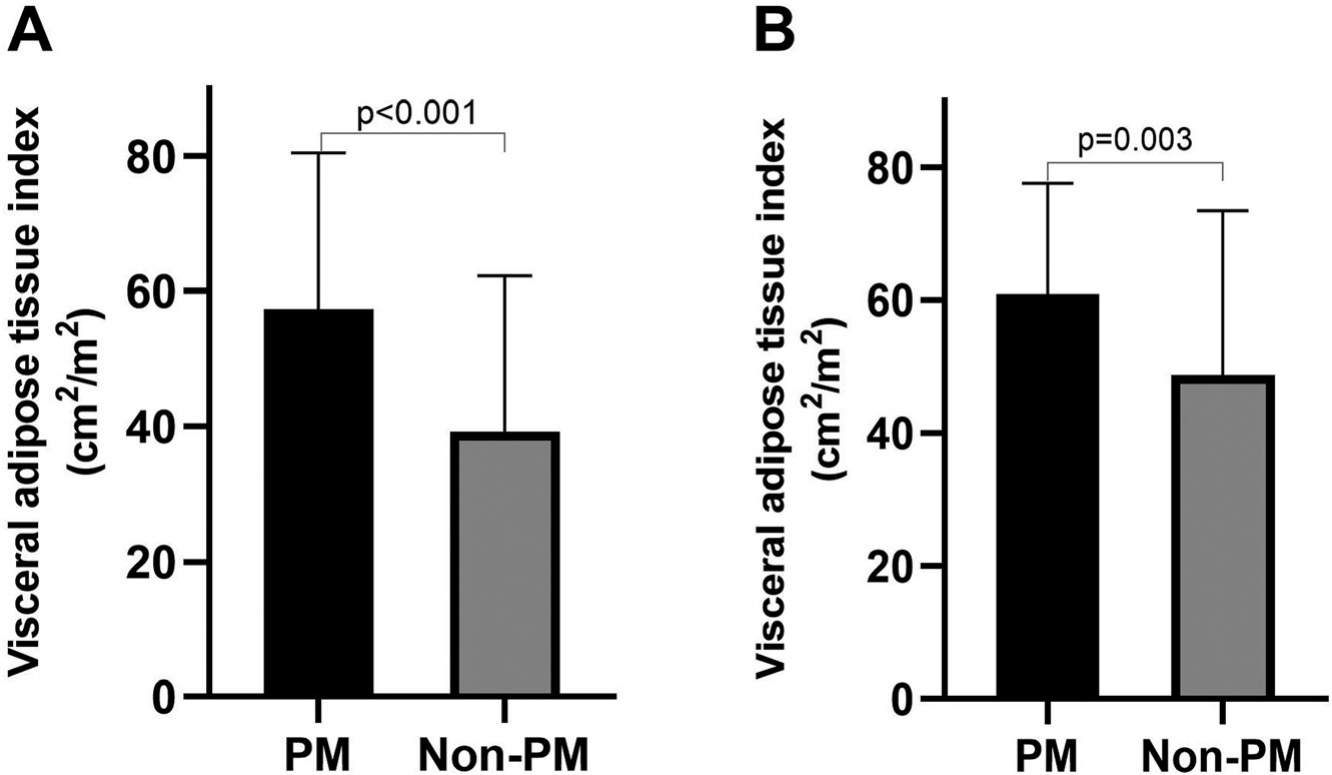
Visceral adipose tissue index (VATI) is significantly elevated in Peritoneal Metastasis (PM) groups. (A) Primary cohort (n=405): Patients with PM exhibited higher VATI compared to non-PM patients (PM: 57.4 ± 23.2 cm^2^/m^2^ vs. non-PM: 39.3 ± 23.0 cm^2^/m^2^; p<0.001, Student’s *t*-test). (B) External validation cohort (n=262): consistent elevation of VATI in PM group (PM: 60.9 ± 16.7 cm^2^/m^2^ vs. non-PM: 48.7 ± 24.7 cm^2^/m^2^; p=0.003). Error bars represent mean ± SD.

VATI was significantly higher in PM patients. In the primary cohort, PM patients had a mean VATI of 57.4 ± 23.2 cm^2^/m^2^ compared to 39.3 ± 23.0 cm^2^/m^2^ in non-PM patients (p<0.001) (Figure 1A). A similar trend was observed in the validation cohort, with PM patients exhibiting higher VATI (55.1 ± 24.7 cm^2^/m^2^ vs. 37.8 ± 21.5 cm^2^/m^2^; p<0.001) (Figure 1B).

### Stratified PM risk by VATI tertiles

**Figure 2:**
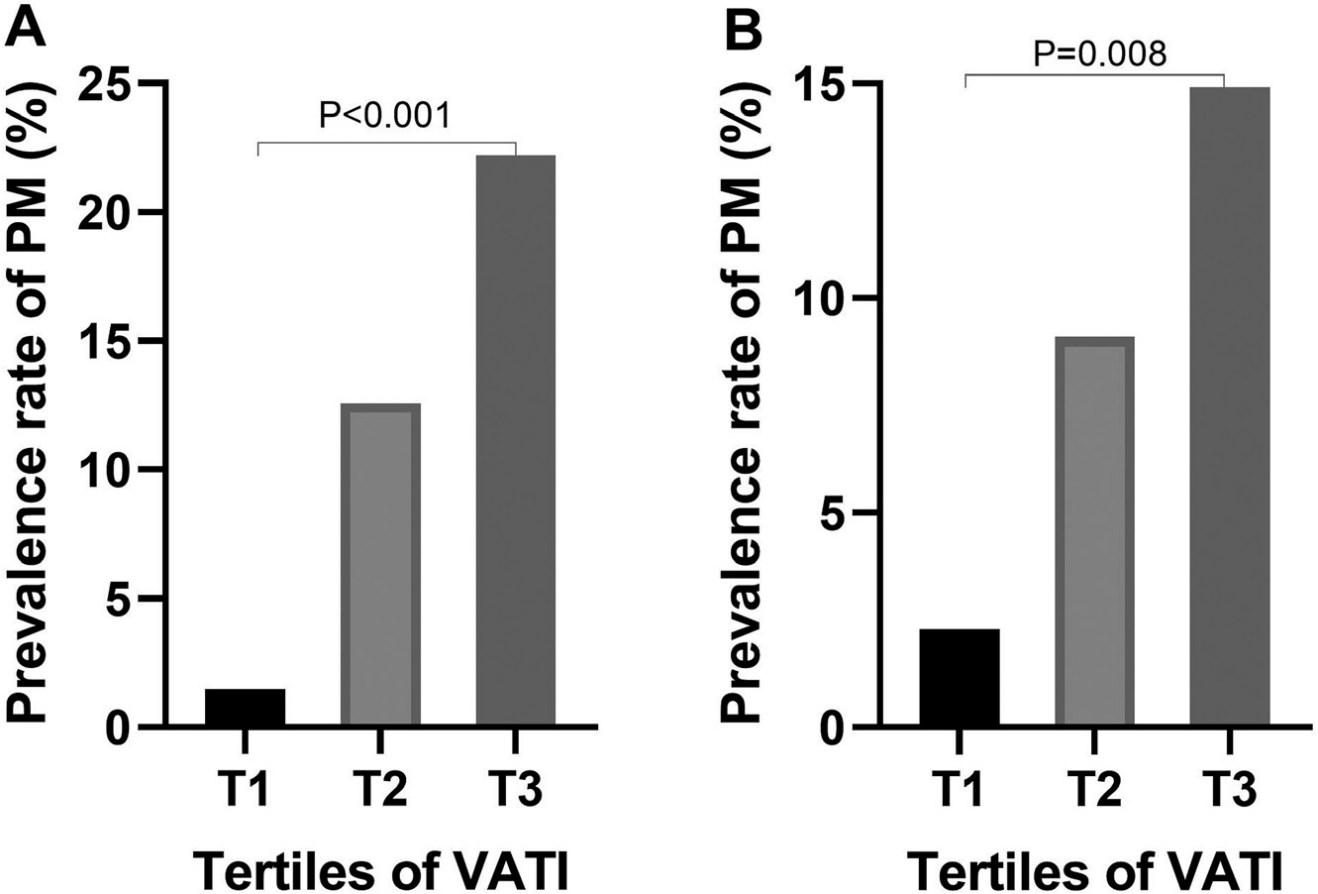
Dose-dependent association between VATI tertiles and Peritoneal Metastasis (PM) risk. (A) Primary cohort: PM prevalence escalated with increasing VATI tertiles (T1 [≤29.92 cm^2^/m^2^]: 1.48 % [2/135]; T2 [29.92–47.95 cm^2^/m^2^]: 12.59 % [17/135]; T3 [≥47.96 cm^2^/m^2^]: 22.22 % [30/135]; trend p<0.001). (B) External validation cohort: replicated dose-response relationship (T1: 3.2 % [1/31]; T2: 15.6 % [5/32]; T3: 25.0 % [8/32]; trend p=0.008).

Patients were stratified into tertiles based on VATI: T1 (≤29.92 cm^2^/m^2^), T2 (29.92–47.95 cm^2^/m^2^), and T3 (≥47.96 cm^2^/m^2^). In the primary cohort, PM prevalence increased markedly across tertiles: T1 (1.48 %, 2/135), T2 (12.59 %, 17/135), and T3 (22.22 %, 30/135; p<0.001) (Figure 2A). The validation cohort mirrored this trend, with PM rates rising from 3.2 % (T1) to 25.0 % (T3; p=0.008), confirming a dose-dependent relationship between VATI and PM risk (Figure 2B).

### Predictive power of VATI

ROC analysis showed that the optimal cutoff value for VATI was 43.58 cm^2^/m^2^ for PM. The specificity and sensitivity were 62.6 and 79.6 %, respectively (AUC=0.736, 95 % CI: 0.690–0.778, p<0.0001) (Figure S2).

### Multivariate regression analysis

In the primary cohort, independent risk factors for PM included albumin [OR (95 % CI)=0.867 (0.797–0.944), p=0.001], mucinous/signet ring cell histology [OR (95 % CI)=2.455 (1.071–5.627), p<0.001], T4 stage [OR (95 % CI)=6.310 (2.250–17.698), p<0.001], N1/2 stage [OR (95 % CI)=6.104 (2.600–14.333), p<0.001], CA19-9≥37 U/mL [OR (95 % CI)=3.262 (1.404–7.578), p=0.006], and high VATI [OR (95 % CI)=1.029 (1.014–1.044), p<0.001]. The validation cohort identified right-sided tumors [OR (95 % CI)=3.038 (1.067–8.653), p=0.037] and CEA≥5 ng/mL [OR (95 % CI)=4.270 (1.352–13.483), p=0.013] as additional predictors, while high VATI [OR (95 % CI)=1.022 (1.001–1.044), p=0.041] remained a consistent risk factor (Tables 2 and 4).

**Table 2:** Logistic regression analysis of peritoneal metastasis in the primary cohort.

Variables	Univariate analysis		p-Value	Multivariate analysis		p-Value
	β	OR (95 % CI)		β	OR (95 % CI)	
Age, years	0.039	1.040 (1.010–1.071)	0.008			
Histological type (mucinous/Signet ring cell vs. adenocarcinoma)	1.355	3.876 (2.028–7.408)	<0.001	0.898	2.455 (1.071–5.627)	0.034
T stage (T4 vs. T1–T3)	2.057	7.825 (3.647–16.788)	0.008	1.842	6.310 (2.250–17.698)	<0.001
N stage (N1/N2 vs. N0)	1.891	6.626 (3.271–13.421)	<0.001	1.809	6.104 (2.600–14.333)	<0.001
CEA, ng/mL (>5 vs.≤5)	0.958	2.605 (1.422–4.773)	0.002			
CA19-9, U/mL (>37 vs.≤37)	1.779	5.927 (3.040–11.554)	<0.001	1.182	3.262 (1.404–7.578)	0.006
Albumin, g/L	−0.152	0.859 (0.804–0.918)	<0.001	−0.142	0.867 (0.797–0.944)	0.001
VATI, cm^2^/m^2^	0.029	1.029 (1.017–1.042)	<0.001	0.029	1.029 (1.014–1.044)	<0.001

T stage, tumor stage; N stage, nodal stage; VATI, visceral adipose tissue index; CEA, carcinoembryonic antigen; CA19-9, carbohydrate antigen 19-9; β, regression coefficient; OR, odds ratio; CI, confidence interval.

**Table 3:** The characteristics of CRC patients according to PM status in the validation cohort.

Variables	Total n=262	PM (+) n=23	PM (−) n=239	p-Value
Age, years	62.6 ± 10.7	61.2 ± 12.3	62.7 ± 12.6	0.510
Gender, %				
Male	162 (61.8)	18 (78.3)	144 (60.3)	0.089
Female	100 (38.2)	5 (21.7)	95 (39.7)	
Tumor location, %				
Right colon	80 (30.5)	13 (56.5)	67 (28.0)	0.005
Left colon + rectum	182 (69.5)	10 (43.5)	172 (72.0)	
Histological type, %				
Adenocarcinoma	208 (79.4)	11 (47.8)	197 (82.4)	<0.001
Mucinous/Signet ring cell	54 (20.6)	12 (52.2)	42 (17.6)	
T stage, %				
T1–T3	241 (92.0)	19 (82.6)	222 (92.9)	0.098
T4	21 (8.0)	4 (17.4)	17 (7.1)	
N stage, %				
N0	170 (64.9)	13 (56.5)	157 (65.7)	0.379
N1/N2	92 (35.1)	10 (43.5)	82 (34.3)	
CEA, ng/mL				
≤5	168 (64.1)	5 (21.7)	163 (68.2)	<0.001
>5	94 (35.9)	18 (78.3)	76 (31.8)	
CA19-9, U/mL				
≤37	230 (87.8)	13 (56.5)	217 (90.8)	<0.001
>37	32 (12.2)	10 (43.5)	22 (9.2)	
Hypertension				
Yes	95 (36.3)	6 (26.1)	89 (37.2)	0.288
No	167 (63.7)	17 (73.9)	150 (62.8)	
Diabetes				
Yes	36 (10.6)	3 (13.0)	33 (13.8)	1.000
No	226 (89.4)	20 (87.0)	206 (86.2)	
Albumin, g/L	42.8 ± 5.2	40.3 ± 3.3	43.1 ± 3.3	0.013
Visceral adipose tissue area, cm^2^	139.1 ± 70.9	173.1 ± 51.2	135.8 ± 71.7	0.003
Visceral adipose tissue index, cm^2^/m^2^	49.8 ± 24.4	60.9 ± 16.7	48.7 ± 24.7	0.003

CRC, colorectal cancer; PM, peritoneal metastasis; T stage, tumor stage; N stage, nodal stage; CEA, carcinoembryonic antigen; CA19-9, carbohydrate antigen 19-9.

**Table 4:** Logistic regression analysis of peritoneal metastasis in the validation cohort.

Variables	Univariate analysis		p-Value	Multivariate analysis		p-Value
	β	OR (95 % CI)		β	OR (95 % CI)	
Gender (male vs. female)	0.865	2.375 (0.853–6.614)	0.098			
Tumor location (left colon+Rectum vs. right colon)	1.592	4.914 (2.035–11.863)	<0.001	1.111	3.038 (1.067–8.653)	0.037
Histological type (mucinous/Signet ring cell vs. Adenocarcinoma)	1.633	5.117 (2.115–12.378)	<0.001	1.731	5.648 (1.939–16.448)	0.002
T stage (T4 vs. T1-T3)	1.011	2.749 (0.840–8.998)	0.095			
CEA, ng/mL (>5 vs.≤5)	2.019	7.532 (2.695–21.050)	<0.001	1.452	4.270 (1.352–13.483)	0.013
CA19-9, U/mL (>37 vs.≤37)	2.008	7.448 (2.927–18.951)	<0.001	1.117	3.055 (1.008–9.256)	0.048
Albumin, g/L	−0.121	0.886 (0.808–0.970)	0.009	−0.118	0.889 (0.792–0.997)	0.044
VATI, cm^2^/m^2^	0.020	1.020 (1.003–1.038)	0.024	0.022	1.022 (1.001–1.044)	0.041

VATI, visceral adipose tissue index; T stage, tumor stage; CEA, carcinoembryonic antigen; CA19-9, carbohydrate antigen 19-9; β, regression coefficient; OR, odds ratio; CI, confidence interval.

## Discussion

Given the significant burden of PM in terms of mortality, PM is traditionally perceived as a terminal condition. Our study identifies VATI as an independent predictor of synchronous PM in CRC, addressing a critical gap in CRC metastasis research. These findings position VATI as a practical biomarker for preoperative risk stratification, enabling targeted interventions such as enhanced imaging (e.g., diagnostic laparoscopy) or prophylactic hyperthermic intraperitoneal chemotherapy (HIPEC) in high-risk patients.

Previous studies have demonstrated that VAT is associated with postoperative complications and overall survival [16], 17]. Excess fat accumulation triggers inflammatory mediator release, establishing a chronic inflammatory state [18]. Within this inflamed tumor microenvironment, hypertrophic adipocytes secrete adipokines and cytokines that promote tumor proliferation, angiogenesis, and progression [19]. VAT overproduces leptin in obesity, activating its receptor on CRC cells. This triggers JAK2/STAT3 signaling, upregulating pro-survival genes (e.g., BCL-2) and epithelial-mesenchymal transition transcription factors (e.g., SNAIL, TWIST) [20]. Moreover, leptin induces blood vessel formation through the PI3K-Akt-mTOR pathway, facilitating peritoneal invasion [21]. On the contrary, adiponectin inhibits CRC proliferation by activating AMPK, suppressing mTOR-driven protein synthesis [15]. Low adiponectin levels in VAT-rich patients depress mTOR, accelerating liver metastasis [22]. Crown-like structures in VAT secrete IL-6 and TNF-α via NF-κB activation [13], 23]. IL-6 binds to IL-6 receptor on CRC cells, activating STAT3 to upregulate CCL2 (macrophage recruitment) [14]. TNF-α promotes Wnt/β-catenin signaling, enhancing stemness and chemoresistance in metastatic CRC [24], 25]. Additionally, the colon’s direct anatomical connection to visceral fat allows tumors to penetrate the serous membrane and invade the omentum/mesentery [26]. Once in the peritoneal cavity, cancer cells form mobile thrombi, degrade extracellular matrix components, and implant into mesothelial tissues, ultimately driving peritoneal metastasis [27]. However, more research is needed to clarify the precise mechanism of VAT in the development of PM in CRC.

Consistent with other results, our study revealed that primary tumor location, CA19-9, and histological type were the independent risk factors for synchronous PM. Right-sided tumors have a higher risk for PM, which has been confirmed by prior studies [28], [29], [30]. Higher pretreatment CA19-9 levels indicate a more advanced TNM stage and are associated with poor pathological type [31]. Furthermore, mucinous/signet ring cell histological type was also consistently identified as a key clinical predictor of synchronous PM [32]. Our preliminary data showed that VATI, combined with tumor markers (CEA and CA19-9), forms a robust predictive model for PM (Figure S3). Further confirmatory research is needed.

Our findings have significant implications for clinical practice. Current screening relies heavily on CT/MRI, which may miss micrometastases. Integrating VATI into preoperative risk stratification could prioritize high-risk patients and optimize surveillance intervals. High-VATI patients may warrant advanced imaging (PET-CT) or diagnostic laparoscopy to detect occult peritoneal deposits and undergo shorter-interval follow-up to improve early detection rates. For high VATI patients undergoing curative resection, hyperthermic intraperitoneal chemotherapy or extended peritoneal lavage should be considered to mitigate metastasis risk. VATI reflects a pro-metastatic adipose microenvironment. Pharmacologic inhibition of VAT-associated mediators may disrupt PM progression. In addition, high VATI correlates with immunosuppressive tumor microenvironment [19], 33]. Earlier introduction of immune checkpoint inhibitors in high-VATI subgroups could be considered in future clinical trials.

This study has several limitations. First, as a retrospective analysis, prospective validation in multiethnic cohorts is needed. Second, although VAT is significant in the development of PM, the underlying mechanisms remain unclear. Finally, some factors such as dietary habits, physical activity, or genetic predispositions could influence VATI and CRC progression [34], [35], [36]. In addition, both gender and ethnicity significantly influence VAT distribution [37]. Therefore, future clinical research should take these possible confounders into account.

## Conclusions

Visceral adipose tissue index independently predicts synchronous peritoneal metastasis in CRC. Our findings advocate for VATI-guided risk stratification while underscoring the need to investigate adipose-driven metastatic mechanisms.

## Supplementary Material

Supplementary Material

## References

[1] Siegel RL, Giaquinto AN, Jemal A (2024). Cancer statistics, 2024. CA Cancer J Clin.

[2] Brouwer NPM, van der Kruijssen DEW, Hugen N, de Hingh IHJT, Nagtegaal ID, Verhoeven RHA (2020). The impact of primary tumor location in synchronous metastatic colorectal cancer: differences in metastatic sites and survival. Ann Surg Oncol.

[3] Kobayashi H, Kotake K, Sugihara K (2014). Outcomes of surgery without HIPEC for synchronous peritoneal metastasis from colorectal cancer: data from a multi-center registry. Int J Clin Oncol.

[4] Kobayashi H, Kotake K, Funahashi K, Hase K, Hirata K, Iiai T (2014). Clinical benefit of surgery for stage IV colorectal cancer with synchronous peritoneal metastasis. J Gastroenterol.

[5] Spiliotis J, Kalles V, Kyriazanos I, Terra A, Prodromidou A, Raptis A (2019). CRS and HIPEC in patients with peritoneal metastasis secondary to colorectal cancer: the small-bowel PCI score as a predictor of survival. Pleura Peritoneum.

[6] Sato H, Toyama K, Koide Y, Ozeki S, Hatta K, Maeda K (2016). Prognoses and treatment strategies for synchronous peritoneal dissemination of colorectal carcinoma. Surg Today.

[7] Dresen RC, De Vuysere S, De Keyzer F, Van Cutsem E, Prenen H, Vanslembrouck R (2019). Whole-body diffusion-weighted MRI for operability assessment in patients with colorectal cancer and peritoneal metastases. Cancer Imaging.

[8] Foster JM, Zhang C, Rehman S, Sharma P, Alexander HR (2023). The contemporary management of peritoneal metastasis: a journey from the cold past of treatment futility to a warm present and a bright future. CA Cancer J Clin.

[9] Kirichenko TV, Markina YV, Bogatyreva AI, Tolstik TV, Varaeva YR, Starodubova AV (2022). The role of adipokines in inflammatory mechanisms of obesity. Int J Mol Sci.

[10] Brown KA, Scherer PE (2023). Update on adipose tissue and cancer. Endocr Rev.

[11] Dumas JF, Brisson L (2021). Interaction between adipose tissue and cancer cells: role for cancer progression. Cancer Metastasis Rev.

[12] Nasser NJ, Fox J, Agbarya A (2020). Potential mechanisms of cancer-related hypercoagulability. Cancers (Basel).

[13] Anderson LJ, Lee J, Anderson B, Lee B, Migula D, Sauer A (2022). Whole-body and adipose tissue metabolic phenotype in cancer patients. J Cachexia Sarcopenia Muscle.

[14] Zhong Q, Fang Y, Lai Q, Wang S, He C, Li A (2020). CPEB3 inhibits epithelial-mesenchymal transition by disrupting the crosstalk between colorectal cancer cells and tumor-associated macrophages via IL-6R/STAT3 signaling. J Exp Clin Cancer Res.

[15] Chakraborty D, Jin W, Wang J (2021). The bifurcated role of adiponectin in colorectal cancer. Life Sci.

[16] Park JW, Chang SY, Lim JS, Park SJ, Park JJ, Cheon JH (2022). Impact of visceral fat on survival and metastasis of stage III colorectal cancer. Gut Liver.

[17] Basile D, Bartoletti M, Polano M, Bortot L, Gerratana L, Di Nardo P (2021). Prognostic role of visceral fat for overall survival in metastatic colorectal cancer: a pilot study. Clin Nutr.

[18] Kwaifa IK, Bahari H, Yong YK, Noor SM (2020). Endothelial dysfunction in obesity-induced inflammation: molecular mechanisms and clinical implications. Biomolecules.

[19] Chaplin A, Rodriguez RM, Segura-Sampedro JJ, Ochogavía-Seguí A, Romaguera D, Barceló-Coblijn G (2022). Insights behind the relationship between colorectal cancer and obesity: is visceral adipose tissue the missing link?. Int J Mol Sci.

[20] Socol CT, Chira A, Martinez-Sanchez MA, Nuñez-Sanchez MA, Maerescu CM, Mierlita D (2022). Leptin signaling in obesity and colorectal cancer. Int J Mol Sci.

[21] Herrera-Vargas AK, García-Rodríguez E, Olea-Flores M, Mendoza-Catalán MA, Flores-Alfaro E, Navarro-Tito N (2021). Pro-angiogenic activity and vasculogenic mimicry in the tumor microenvironment by leptin in cancer. Cytokine Growth Factor Rev.

[22] Świerczyński M, Szymaszkiewicz A, Fichna J, Zielińska M (2021). New insights into molecular pathways in colorectal cancer: adiponectin, interleukin-6 and opioid signaling. Biochim Biophys Acta Rev Cancer.

[23] Bruno MEC, Mukherjee S, Powell WL, Mori SF, Wallace FK, Balasuriya BK (2022). Accumulation of γδ T cells in visceral fat with aging promotes chronic inflammation. Geroscience.

[24] Zhao X, Ma L, Dai L, Zuo D, Li X, Zhu H (2020). TNF-α promotes the malignant transformation of intestinal stem cells through the NF-κB and Wnt/β-catenin signaling pathways. Oncol Rep.

[25] Novoa Díaz MB, Martín MJ, Gentili C (2022). Tumor microenvironment involvement in colorectal cancer progression via Wnt/β-catenin pathway: providing understanding of the complex mechanisms of chemoresistance. World J Gastroenterol.

[26] Tabuso M, Homer-Vanniasinkam S, Adya R, Arasaradnam RP (2017). Role of tissue microenvironment resident adipocytes in colon cancer. World J Gastroenterol.

[27] Xia W, Geng Y, Hu W (2023). Peritoneal metastasis: a dilemma and challenge in the treatment of metastatic colorectal cancer. Cancers (Basel).

[28] Hutchins G, Southward K, Handley K, Magill L, Beaumont C, Stahlschmidt J (2011). Value of mismatch repair, KRAS, and BRAF mutations in predicting recurrence and benefits from chemotherapy in colorectal cancer. J Clin Oncol.

[29] Benedix F, Kube R, Meyer F, Schmidt U, Gastinger I, Lippert H (2010). Comparison of 17,641 patients with right- and left-sided colon cancer: differences in epidemiology, perioperative course, histology, and survival. Dis Colon Rectum.

[30] Glebov OK, Rodriguez LM, Nakahara K, Jenkins J, Cliatt J, Humbyrd CJ (2003). Distinguishing right from left colon by the pattern of gene expression. Cancer Epidemiol Biomarkers Prev.

[31] Mo S, Dai W, Xiang W, Li Q, Wang R, Cai G (2018). Predictive factors of synchronous colorectal peritoneal metastases: development of a nomogram and study of its utilities using decision curve analysis. Int J Surg.

[32] Kermanshahi TR, Magge D, Choudry H, Ramalingam L, Zhu B, Pingpank J (2017). Mucinous and signet ring cell differentiation affect patterns of metastasis in colorectal carcinoma and influence survival. Int J Surg Pathol.

[33] Zhu C, Teng L, Lai Y, Yao X, Fang Y, Wang Z (2024). Adipose-derived stem cells promote glycolysis and peritoneal metastasis via TGF-β1/SMAD3/ANGPTL4 axis in colorectal cancer. Cell Mol Life Sci.

[34] Martínez-Montoro JI, Martínez-Sánchez MA, Balaguer-Román A, Gil-Martínez J, Mesa-López MJ, Egea-Valenzuela J (2022). Dietary modulation of gut microbiota in patients with colorectal cancer undergoing surgery: a review. Int J Surg.

[35] McGettigan M, Cardwell CR, Cantwell MM, Tully MA (2020). Physical activity interventions for disease-related physical and mental health during and following treatment in people with non-advanced colorectal cancer. Cochrane Database Syst Rev.

[36] Zaborowski AM, Abdile A, Adamina M, Aigner F, d’Allens L, Allmer C (2021). Characteristics of early-onset vs late-onset colorectal cancer: a review. JAMA Surg.

[37] Kastrinos F, Kupfer SS, Gupta S (2023). Colorectal cancer risk assessment and precision approaches to screening: brave new world or worlds apart?. Gastroenterology.

