# Supplementary material for: Visceral adipose tissue is associated with occult synchronous peritoneal metastasis in colorectal cancer
Source: Pleura Peritoneum. 2025 Apr 24;10(2):81–8. doi: 10.1515/pp-2024-0030 (PMC12207390; doi:10.1515/pp-2024-0030)

**Figure S1.** Study Design and Cohort Selection Workflow. Schematic overview of patient inclusion/exclusion criteria.


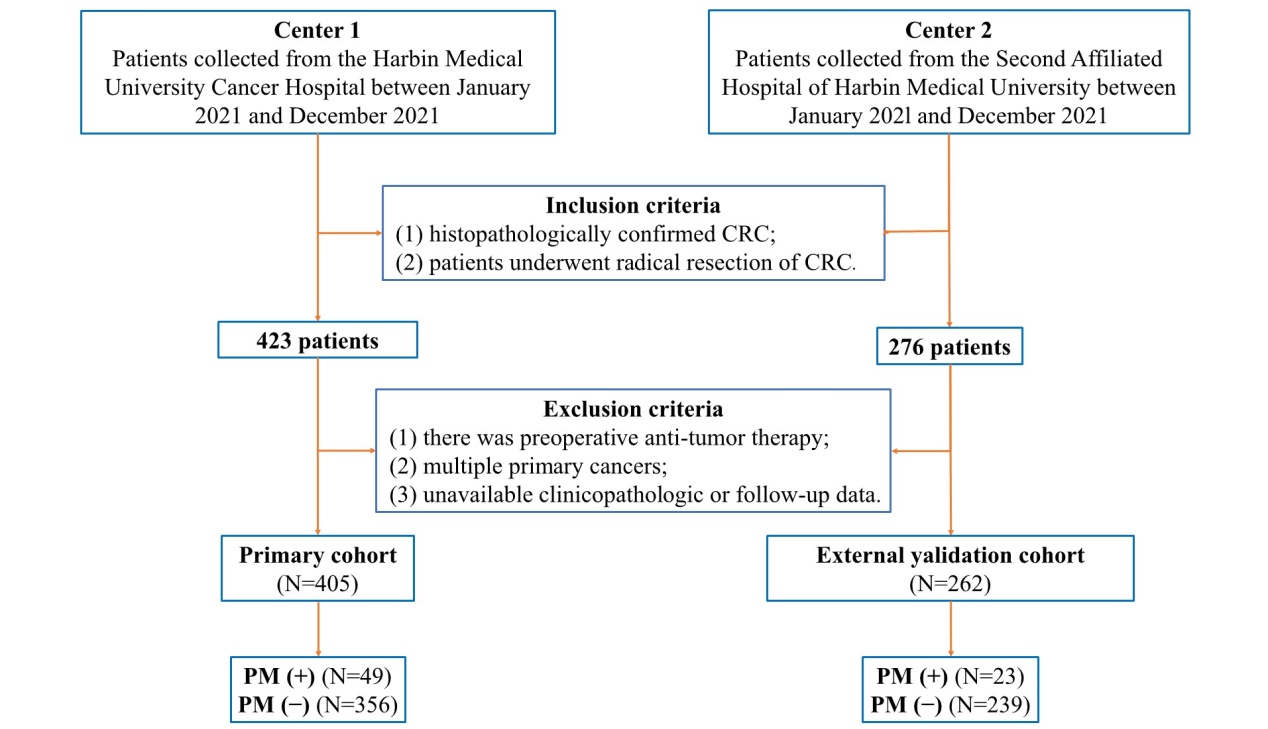


**Figure S2.** An optimized cut-off value was calculated for VATI using ROC curve analysis. The ROC curve identified the optimal cutoff value of VATI as 43.58 cm^2^/m^2^.


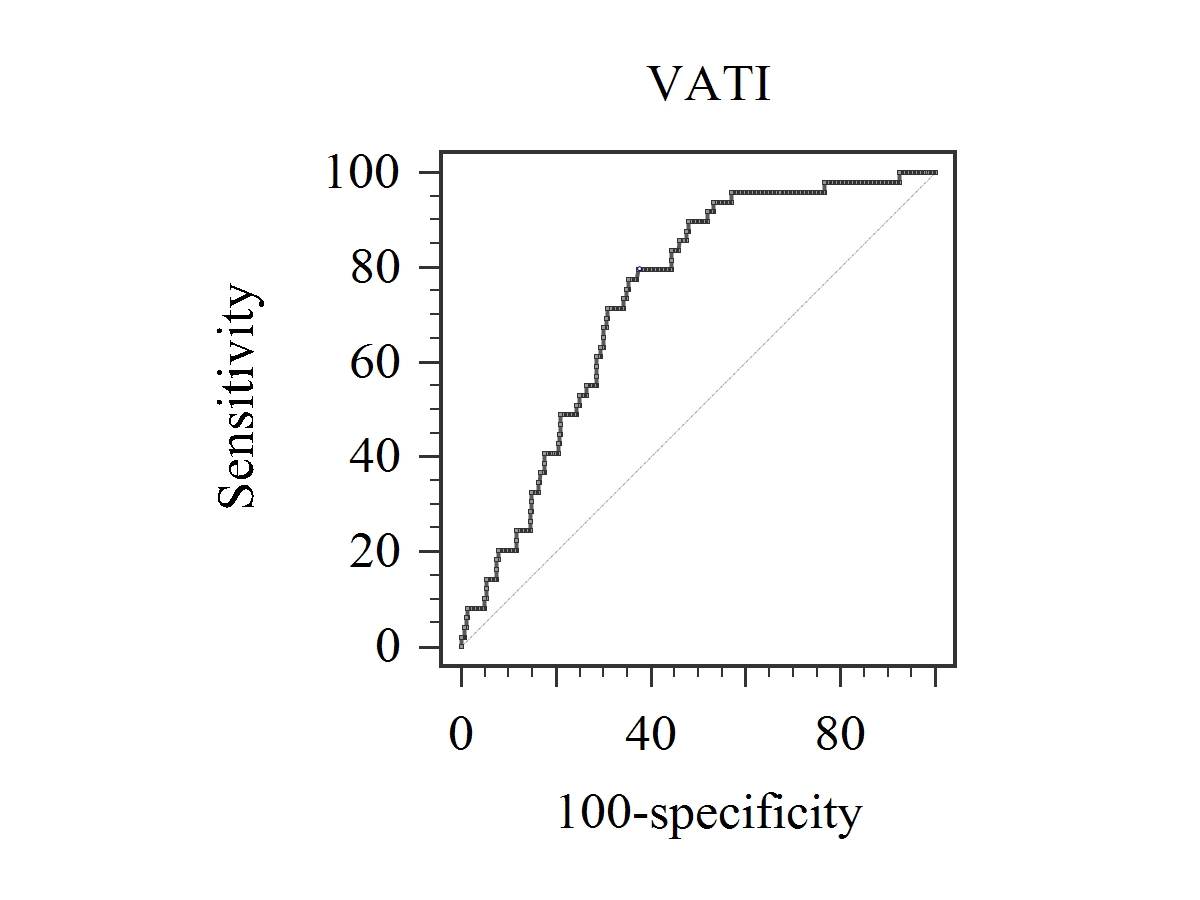


**Figure S3.** The discriminative performance of single or combined predictors. After adding VATI to the model (CEA+CA199+VATI), the model dramatically improved the discriminative efficacy compared to the model (CEA+CA199) (p=0.023).


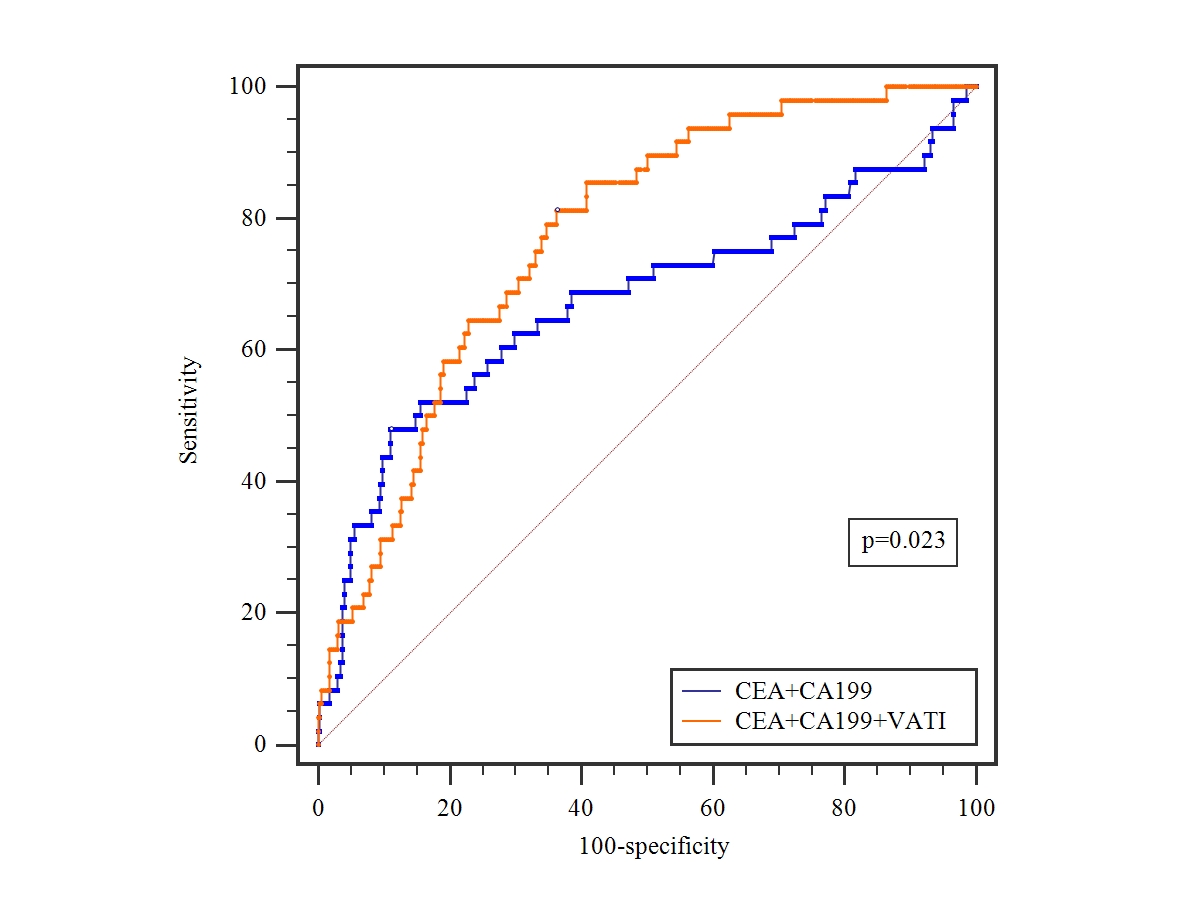

Supplement: Supplementary file 1 — Supplementary Material [file j_pp-2024-0030_suppl_001.docx]
